# Recent Progress in Pharmaceutical Therapies for Castration-Resistant Prostate Cancer

**DOI:** 10.3390/ijms140713958

**Published:** 2013-07-04

**Authors:** Lina Yin, Qingzhong Hu, Rolf W. Hartmann

**Affiliations:** Pharmaceutical and Medicinal Chemistry, Saarland University & Helmholtz Institute for Pharmaceutical Research Saarland (HIPS), Campus C2-3, Saarbrücken D-66123, Germany; E-Mail: linayin98@hotmail.com

**Keywords:** castration-resistant prostate cancer, Abiraterone, Enzalutamide, Cabazitaxel, Sipuleucel-T, Denosumab, Alpharadin

## Abstract

Since 2010, six drugs have been approved for the treatment of castration-resistant prostate cancer, *i.e.*, CYP17 inhibitor Abiraterone, androgen receptor antagonist Enzalutamide, cytotoxic agent Cabazitaxel, vaccine Sipuleucel-T, antibody Denosumab against receptor activator of nuclear factor kappa B ligand and radiopharmaceutical Alpharadin. All these drugs demonstrate improvement on overall survival, expect for Denosumab, which increases the bone mineral density of patients under androgen deprivation therapy and prolongs bone-metastasis-free survival. Besides further CYP17 inhibitors (Orteronel, Galeterone, VT-464 and CFG920), androgen receptor antagonists (ARN-509, ODM-201, AZD-3514 and EZN-4176) and vaccine Prostvac, more drug candidates with various mechanisms or new indications of launched drugs are currently under evaluation in different stages of clinical trials, including various kinase inhibitors and platinum complexes. Some novel strategies have also been proposed aimed at further potentiation of antitumor effects or reduction of side effects and complications related to treatments. Under these flourishing circumstances, more investigations should be performed on the optimal combination or the sequence of treatments needed to delay or reverse possible resistance and thus maximize the clinical benefits for the patients.

## 1. Introduction

The aging of population increases the incidence of prostate cancer (PCa) because the median age of PCa patients being diagnosed is 67. Estimation has been made that in the US nearly a quarter-million PCa cases emerged in 2012 accounting for 29% of the total new cancer cases [1]. In contrast, 28,170 patients died of PCa composing only 9% of the total cancer related death [1]. This difference is probably because most of the PCa patients were identified in the early stages and therefore could be cured with local therapies, such as prostatectomy, radiation and cryotherapy, with the five-year survival approaching 100% [2]. However, for advanced metastatic cases these treatments show little benefit and without effective control the patients ineluctably die. Androgen deprivation and chemotherapy are currently standard treatments for these patients. However, after long term application nearly all patients are no longer sensitive to these treatments. Progression or relapse happen even under the circumstance that the plasma concentrations of testosterone are suppressed to around 50 ng/dL by castration or gonadotropin-releasing hormone (GnRH) analogues, while the effects of the remaining androgens are blocked by androgen receptor (AR) antagonists. This situation is termed as castration-resistant prostate cancer (CRPC), which has been mis-comprehended as “androgen independent”. However, recent research revealed that CRPC is still driven by hormones [3]. Many possible mechanisms have been proposed for CRPC, such as super-sensitivity of tumor cells to low levels of androgen, intratumoral androgen para-/autocrine production, AR mutation and ligand independent AR activation. Since 2010, six drugs have been approved for the treatment of CRPC. These drugs either show improvement on overall survival or relieve the symptoms regarding bones, which are the most frequent sites that metastases happen due to the abundant growth factors facilitating the proliferation of cancer cells [4]. More drug candidates are currently under evaluation in different stages of clinical trials and some novel strategies have also been proposed aimed at further potentiation of antitumor effects or reduction of side effects and complications related to treatments.

## 2. CYP17 Inhibitor Abiraterone

**Scheme 1:**
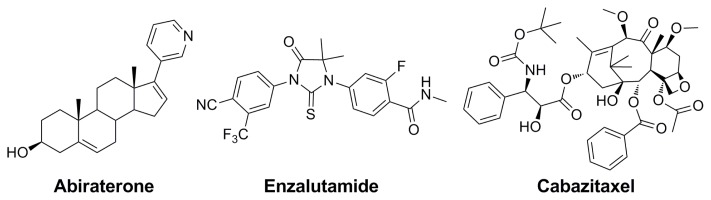
Structures of Abiraterone, Enzalutamide and Cabazitaxel.

Hormone therapy is based on the observation that up to 80% of PCa proliferation is stimulated by androgens [5]. Therefore, orchiectomy or GnRH analogues (chemical castration) have been employed to reduce androgen levels because around 90% of androgens are produced in testes. However, it is apparent that they cannot prevent the biosyntheses of androgen inside adrenals and tumor cells. This leak results in sufficient androgens inside the prostate to continue stimulating PCa cells [6]. In contrast, the inhibition of 17α-hydroxylase-17,20-lyase (CYP17) is a superior approach to eradicate androgen secretion because this enzyme is pivotal in androgen biosynthesis regardless of production location. Abiraterone (Scheme 1) is the first CYP17 inhibitor, launched in 2011 as the acetate prodrug. It competitively inhibits the enzyme via the coordination of its *sp*^2^ hybrid pyridyl N to heme iron, which is the reactive centre of CYP enzymes for activating O_2_ and oxidizing the substrates. Due to the common catalytic mechanism across the CYP family, this inhibitory artifice has been successfully exploited in not only CYP17 inhibitors [7–14], but also inhibitors of other steroidogenic CYP enzymes, e.g., aromatase (CYP19) [15–19], 11β-hydroxylase (CYP11B1) [20–22] and aldosterone synthase (CYP11B2) [23–29]. Abiraterone significantly inhibits both activities of CYP17, *i.e.*, 17α-hydroxylase and C17-20 lyase, with similar potency (IC_50_ of 4 and 2.9 nM, respectively) in a human testicular microsome assay [30]. This potent inhibition leads to a reduction of the plasma testosterone concentrations to less than 1 ng/dL [3]. However, the plasma levels of 11-deoxycorticosterone (DOC) and corticosterone are boosted by 10- and 40-fold, respectively [18], which result from the inhibition of 17α-hydroxylase activity and CYP11B1 [31] via a 2-fold decrease of cortisol concentration and the subsequent 5-fold promotion of ACTH secretion [3]. The elevation of mineralocorticoids leads to hypokalemia, hypertension and edema, which have to be suppressed by the co-application of Prednisone. On the other hand, Abiraterone not only eliminates around 80% of the steady-state AR in LNCaP cells, but also blocks the AR mediated transactivation [32]. This antagonism of AR may also contribute to the anti-tumor effects. When Abiraterone is orally applied as an acetate prodrug (1000 mg per day), the release of free Abiraterone is very rapid and thorough so that Abiraterone reaches maximum plasma concentration (226 ± 178 ng/mL) in 2 h [33], whereas Abiraterone acetate is undetectable in plasma [33,34]. In contrast, approximately half of the applied Abiraterone is excreted as unchanged Abiraterone acetate via feces [33,35], which might indicate a poor gastrointestinal absorption. Around 70% of patients, who are naïve to Ketoconazole and Docetaxel, show at least 50% decline of prostate specific antigen (PSA) levels [36–38], which is a major biomarker in the treatment of CRPC; While for the patients with previous treatments of these two drugs, the response ratio is significantly lower [36–39]. The same trend is also observed for the delay of PSA regression [33–39]. Furthermore, in half of the CRPC patients, the counts of circulating tumor cells are decreased to less than 5 per 7.5 mL blood after Abiraterone treatment [37,38,40]. Symptoms like pain [41,42], pathological fracture [42], spinal cord compression [42] and fatigue [43] are significantly improved as well. More important is the improvement of survival. In post-Docetaxel metastatic CRPC patients, Abiraterone prolongs the median overall survival (15.8 months) by 4.6 months compared to placebo [44,45]. Accordingly, the median radiologic progression-free survival is also improved to 5.6 months, which is around 2 months longer than that of the placebo arm [44,45]. Echoing the better PSA response of Docetaxel-naïve cohort, Abiraterone exhibits a longer median radiologic progression-free survival of 16.5 months in chemotherapy-naïve CRPC patients, which doubles that of the control group (8.3 months) [41]. The initiation of chemotherapy is also delayed by 8.4 months [41]. Due to these apparent benefits, the clinical trial was un-blinded after the interim analysis and all patients were crossed to the Abiraterone group. Although the median overall survival in the Abiraterone arm was therefore not reached in a median follow-up period of 22.2 months, it is clearly longer that of the placebo group (27.2 months) [41].

## 3. AR Antagonist Enzalutamide

The stimulation of androgens on PCa cancer cells is mediated by AR, which is an intracellular transcription factor floating in cytoplasm. The binding of androgen disassociates AR from heat shock proteins and triggers homodimerization, phosphorylation and subsequent translocation into the nucleus. After binding to the androgen response element on DNA, transcription cofactors are recruited to initiate the transcription of the corresponding genes leading to mitogenic effects. Besides, activated AR also interacts directly with various kinase signaling pathways [46,47] promoting the survival and proliferation of PCa cells. AR antagonism is therefore an elegant way of PCa treatment and is employed in combination with GnRH analogues to compose combined androgen blockade. However, after the progression to CRPC, in which AR is highly over-expressed [48], the first generation AR antagonists, such as Flutamide and Bicalutamide, are impotent largely because of the relatively lower affinity compared to dihydrotestosterone (DHT)—the natural substrate of AR and the most potent androgen. These drugs also exhibit partial agonism activity [48] demonstrated by the withdrawal effect observed in the antiandrogen therapy [49]. Moreover, around 15%–30% of patients, who have received long term antiandrogen therapy, develop AR mutations [50], in particular W741C, which recognizes Bicalutamide as an agonist [51]. Fortunately, the second generation AR antagonist Enzalutamide that was launched in 2012 avoids these problems. Enzalutamide exhibits an 8-fold higher AR affinity than Bicalutamide and shows no agonism in the presence of W741C mutation and highly over-expressed AR [52]. Forkhead box transcription factor A, which presets chromatin and thus facilitates the binding of activated AR, only slightly attenuates the antagonism of Enzalutamide to AR, whereas it totally corrupts that of Bicalutamide [53]. Besides the antagonism of AR, including competing with androgens, inhibiting AR translocation into nucleus and impairing its DNA binding and co-activator recruiting, Enzalutamide also induces apoptosis of VCaP cells that exhibit amplified AR gene [52]. In a LNCaP/AR xenograft model in castrated male mice, Enzalutamide led to significant tumor regression [52]. The antitumor effects were further evidenced in clinical trials. More than half of patients under Enzalutamide treatment show serum PSA declines of over 50% [54,55]. The counts of circulating tumor cells as another important biomarker are also turned favorably in around 50% of patients [54]. Enzalutamide exhibits a 6-fold higher soft-tissue response rate compared to the placebo, improves life quality and delays PSA progression and emergence of the first skeletal-related event [55]. These benefits are caused by the on-target effects, *i.e.*, the antagonism of AR, as demonstrated directly by positron emission tomography (PET) imaging with ^18^F-fluoro-5α-dihydrotestosterone [54]. Although seizure is observed in some patients, Enzalutamide is generally tolerable with the most common adverse effect of dose-dependent fatigue [55]. More importantly, the risk of death is reduced by 37% in the CRPC patients having previously received chemotherapy with the median overall survival of 18.4 months [55]. The median radiographic progression-free survival is improved accordingly to 8.3 months as well [55]. Currently, another phase III clinical trial is ongoing on Docetaxel-naïve CRPC patients and the results are eagerly expected.

## 4. Cytotoxic Cabazitaxel

Until 2010, Docetaxel was the last defense line for CRPC and the only drug demonstrating survival benefits [56]. Patients showing no response or progression after Docetaxel therapy can only be managed with Prednisone and Mitoxantrone for palliative purpose. Cabazitaxel, in contrast, is the first agent to improve overall survival in post-Docetaxel patients [57]. As a member of the taxane class, Cabazitaxel unfolds the cytotoxic activity via the same mechanism as Docetaxel by binding to the tubulin to inhibit microtubule depolymerization and thus arresting mitosis. However, Cabazitaxel shows much less affinity to the ATP-dependent drug efflux pump P-glycoprotein (P-gp1) compared to Docetaxel [58]. Since P-gp1 is responsible for multi-drug resistance, this low affinity probably contributes to the superiority of Cabazitaxel over Docetaxel. Cabazitaxel exhibits a terminal half-life of 77 h after intravenous application. It is metabolized in liver by CYP3A4, CYP3A5 and CYP2C8 [58]. Co-application of the inducers or inhibitors of these enzymes should therefore be avoided. Grade 4 neutropenia as the dose limiting toxicity has been observed at 25 mg/m^2^ every 3 weeks [58], hence this dose was applied in the following phase III clinical trials. In post-Docetaxel CRPC patients, the clinical efficacy of Cabazitaxel plus Prednisone was compared to that of the combination of Mitoxantrone and Prednisone. An improved overall survival of 15.1 months was demonstrated in patients under Cabazitaxel treatment in contrast to 12.7 months in the Mitoxantrane arm within a median follow-up of 12.8 months [58]. Moreover, the median progression-free survival (2.8 months), the PSA response ratio (39%) and the time to PSA progression (6.4 months) are twice as high as those of the Mitoxantrane group. However, since normal cells undergoing rapid mitosis are also sabotaged by cytotoxic agents, it is not surprising to observe hematological and gastrointestinal disorders (neutropenia, leukopenia, thrombocytopenia, diarrhea, nausea, and vomiting) [58] as side effects of Cabazitaxel. After balancing the benefits and risks, Cabazitaxel was approved by the FDA. However, it is not considered as a cost-effective therapy due to the incremental costs concerning adverse events and therefore it is not recommended by the National Institute for Health and Clinical Excellence [59].

## 5. Vaccine Sipuleucel-T

Sipuleucel-T, launched in 2010, is mainly a set of antigen-presenting cells (APC) generated from the patient’s own hematopoietic progenitor cells. These APC are loaded with a fusing protein (PA2024) that consists of prostate acid phosphatase (PAP) conjugated with granulocyte-macrophage colony-stimulating factor (GM-CSF). After being infused back into the patients, Sipuleucel-T binds to the T cell receptors on the immature CD8^+^ cytotoxic T lymphocytes and subsequently endows these cells with PAP epitopes, which can further guide them to demolish PCa cells. As a vaccine, Sipuleucel-T also activates the helper CD4^+^ T lymphocytes, which not only attack the cancer cells directly, but also maintain other cytotoxic T lymphocytes via cytokine secretion. Depending on the initial leukapheresis, other cells, such as T cells, B cells and natural killer cells, can also be present in the final product [60]. Since PAP is employed as the navigating epitope, the expression of PAP in at least 25% of cancer cells is a premise. This personalized treatment invokes both humoral and T cell immune response. The IgM and IgG antibodies against PA2024 and PAP are augmented (titers exceeding 400) in 66.2% and 28.5% of the treated patients, both of which are around 20-fold higher compared to those ratios in the placebo group [61]. Similarly, T-cell proliferation responses to PA2024 and PAP were observed to be much more frequent in Sipuleucel-T six weeks after the infusion [61]. After pooling the data obtained from 3 phase III clinical trials [61–63], Sipuleucel-T demonstrates a 26.5% reduction in risk of death and accordingly an improvement of median overall survival of 4.1 months [60]. However, since the time to disease progression (radiographic or clinical events) as the primary end point do not show significant difference between Sipuleucel-T and placebo groups [61–63], the application of other medications, such as Docetaxel, might have an impact on the survival data.

## 6. RANKL Antibody Denosumab

Skeleton is the site most frequently showing PCa metastases. In CRPC patients, the incidence of bone metastases is nearly 90% [64]. These metastatic cancer cells promote bone resorption to release various growth factors that have been stored inside the bones in immobilized forms to stimulate their proliferation, such as platelet-derived growth factors, insulin-like growth factors, transforming growth factor and fibroblast growth factors [65]. Bone metastases cause severe symptoms, e.g., pain, pathologic fractures, spinal cord compression and hypercalcemia, which not only impair life quality, but also can be life-threatening. Moreover, long term application of androgen deprivation therapy (ADT) suppresses androgens and estrogens leading to the decline of bone mineral density (BMD) and an increased risk of fragility fractures. All these events are mediated via interrupting the balance of bone resorption and formation, in which the receptor activator of nuclear factor kappa B (RANK) and its ligand (RANKL) play important roles. RANKL expressed by both osteoblast and activated T cells stimulates the differentiation of osteoclast precursors after binding to the RANK on their membranes. Via activating the nuclear factor kappa B and Jun N-terminal kinase pathways, it further maintains the mature osteoclast cells, facilitates their adherence and ultimately augments bone resorption. It is notable that PCa cells in bone metastases but not in other sites also express RANKL [66]. Denosumab is a monoclonal antibody against RANKL approved in 2010 for bone related complications. In PCa patients under ADT with high fracture risks, it significantly increases the BMD at the lumbar spine (6.7%), total hip (4.8%) and whole body (4.0%) after 24 months [67]. This improvement is observed as early as one month after the application [67]. Accordingly, the total incidence of new vertebral fracture over a duration of three years (3.9%) is reduced by 62% compared to that in the placebo group [67]. Biomarkers of bone turnover, such as serum C-telopeptide, procollagen type I N-terminal peptide and TRAP-5b, are decreased as well [67]. Furthermore, Denosumab is superior to Zoledronic acid in preventing skeletal-related events (SRE) in CRPC patients with bone metastases (time to the first SRE of 20.7 *vs.* 17.1 months, respectively) [68]. It also causes stronger declines in the concentrations of urinary n-telopeptide and serum bone specific alkaline phosphatase, but no difference in PSA levels, investigator-reported disease progression and overall survival compared to Zoledronic acid [68]. These benefits are also observed in post-bisphosphonates patients [69]. More exciting is that Denosumab significantly improves the bone-metastasis-free survival to 29.5 months, which is 4.2 months longer than that of the placebo arm [70]. The time to the first bone metastasis is delayed to 33.2 months as well (*vs.* 29.5 months for placebo group) [70].

## 7. Radiopharmaceutical Alpharadin

Another drug targeting PCa bone metastases is Alpharadin (Xofigo, ^233^RaCl_2_), which was approved by the FDA on 15 May 2013. Alpharadin is a radiopharmaceutical and hence is more suitable for patients with multifocal bone diseases compared to external-beam radiation therapy. Although several radiopharmaceuticals have been employed in the clinic, such as Strontium-89, Samarium-153 and Rhenium-186, their major benefit is just pain palliation. In contrast, Alpharadin is the first and only radiopharmaceutical that demonstrates improvement on overall survival [71]. As a calcium mimic, Alpharadin can be maximally uptaken by the skeletal (40%–60% of the applied dose) after intravenous injection [72]. A ten-fold less amount is found in the red marrow compared to the bones and the distribution in other organs, such as brain, kidneys and adrenals, is very low [73]. Furthermore, Alpharadin mainly emits α-rays, which shows a track length shorter than 100 μm (about 2–10 cell diameters). All these special features added up, render alpharadin as causing less damage to normal tissues, especially bone marrow, than other radiopharmaceuticals and other radiation therapies. Alpharadin not only relieves pain in CRPC patients with bone metastasis, but also postpones the median time to first SRE by three weeks [71]. It reduces the median bone levels of alkaline phosphatase by 65.6%, which, in contrast, are increased by around 9% in the placebo arm [71]. Accordingly, PSA progression is delayed to 26 weeks in contrast to eight weeks in the control group [71]. The most significant achievement is the improvement of overall survival by 3.6 months [74], which has not been seen for other radiopharmaceuticals.

## 8. Other Drug Candidates in Clinical Trials and Novel Strategies

**Scheme 2:**
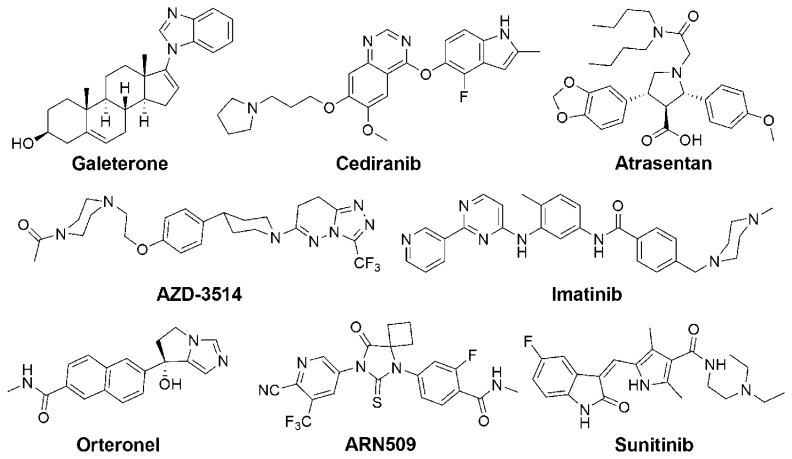

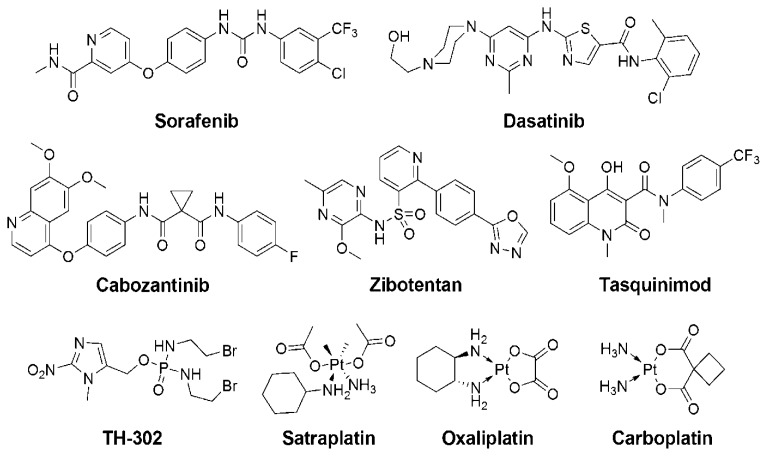
Structures of small molecule drug candidates in clinical trials for CRPC.

Besides these drugs approved recently, there are more drug candidates or new indications of launched drugs under evaluation in clinical trials (Table 1). Galeterone (Scheme 2) is a CYP17 inhibitor but also shows AR antagonism activity. It down-regulates the expression of both wild type and mutated AR, blocks the AR nuclear translocation and the subsequent transcription [75]. A phase I clinical trial revealed PSA responses in around 20% of the patients and the according tumor regression [76]. VT-464 (Scheme 2) is claimed to be a selective inhibitor of C17-20 lyase (one activity of CYP17) and is expected to avoid the secondary mineralocorticoid excess observed for Abiraterone. Experiments in rhesus monkeys confirm that it shows little influence on the concentrations of mineralocorticoids and glucocorticoids [77,78]. Its clinical trial results are expected to show whether this success can be translated into humans. Different from other cytotoxic agents, TH-302 (Scheme 2) is a prodrug specifically activated by hypoxia, which is a common feature inside tumors, but not normal tissues. Fewer side effects are therefore expected than observed after other chemotherapies [79]. PROSTVAC-VF, as a pox viral vaccine expresses PSA and three T-cell co-stimulatory molecules, *i.e.*, B7.1, intercellular adhesion molecule-1 and leukocyte function-associated antigen-3. Due to the employment of viral vectors (vaccinia and fowlpox viruses), potent immune responses are induced and APCs are thus endowed with PSA epitopes. These APCs subsequently activate CD8^+^ cytotoxic T lymphocytes and helper CD4^+^ T lymphocytes, which further attack PCa cells. The application of PROSTVAC-VF not only inhibits the proliferation of cancer cells, but also significantly reduces the tumor growth rate [80]. The latter may explain a phenomenon often observed with vaccines that improved overall survival is not accompanied by a delayed time to progression. Besides cancer vaccines, the blockade of immune checkpoints, e.g., cytotoxic T-lymphocyteassociated antigen-4 (CTLA-4) and programmed death 1 (PD-1), is another promising strategy being extensively investigated. These immune checkpoints are expressed on activated T-cells and serve as modulators to reduce and terminate immuno responses. This physiological function, however, can potentially be exploited by tumor cells to develop resistance [81]. Currently, anti-CTLA-4 antibody Ipilimumab [82] and anti-PD-1 antibody Nivolumab [83] are under evaluation in clinical trials and promising results are reported. Moreover, a heat shock protein 27 inhibitor (OGX-427), various kinase inhibitors, platinum complexes and other entities with different mechanisms are currently also being evaluated as treatments for CRPC (Table 1).

Furthermore, some AR mutations induced by the long term application of ADT have been identified that can be activated by glucocorticoids, in particular cortisol, and thus lead to resistance [103]. Dual inhibition of CYP17/CYP11B1, which is responsible for the biosynthesis of cortisol, is therefore proposed as a novel strategy for the PCa patients with such mutated AR [104]. ADT is also associated with increased cardiovascular mortality [105], while the incidence of cardiac disorders is elevated under Abiraterone treatment as well [41,42]. This is mediated by exorbitant aldosterone, which is a consequence of androgen deficiency caused via various mechanisms, such as the increase of serum low- and high-density lipoprotein (reviewed in reference 106). Since CYP11B2 is the crucial enzyme in the production of aldosterone, dual inhibition of CYP17/CYP11B2 is proposed to reduce the risks of cardiovascular diseases in PCa patients [106].

## 9. Conclusions

Six drugs have been launched for the treatment of castration-resistant prostate cancer since 2010, *i.e.*, CYP17 inhibitor Abiraterone, AR antagonist Enzalutamide, cytotoxic agent Cabazitaxel, vaccine Sipuleucel-T, RANKL antibody Denosumab and radiopharmaceutical Alpharadin. These drugs demonstrate improvement on the overall survival or symptoms relief and prolongation of bone-metastasis-free survival. More drug candidates with various mechanisms or new indications of launched drugs are currently under evaluation in clinical trials. Novel strategies are also proposed to further potentiate the antitumor effects or reduce treatment related side effects and complications. This progress gives CRPC patients hope for a longer life and better life quality. However, resistance to Abiraterone [107] and Enzalutamide [108] has been observed within 12–36 months after initiating the therapies. This resistance to hormone therapy is probably mediated by ligand-independent AR activation and up-regulation of AR and steroidogenic enzymes. Therefore, more investigations should be performed on the optimal combination or the sequence of treatments needed to delay or reverse the resistance and thus maximize the clinical benefits for the patients.

## Figures and Tables

**Table 1:** Drug candidates for the treatment of castration-resistant prostate cancer (CRPC).

Entity a	Category b	Mechanism c	Clinical Trials d
Orteronel [84] (TAK700)	SM	CYP17 inhibitor	**phase I/II** (NCT01666314, NCT01549951[P], NCT00569153, NCT01816048, NCT01658527[B], NCT01046916, NCT01084655[DP]) **Phase III** (NCT01707966, NCT01193257[P], NCT01809691[G]; NCT01546987[BG])
Galeterone [75,76] (TOK-001)	SM	CYP17 inhibitor with AR antagonism	**phase I/II** (NCT00959959#, NCT01709734)
VT-464 [77,78]	SM	selective CYP17 C17-20 lyase inhibitor	**phase I/II** (2011-004103-20) e
CFG920	SM	CYP17 inhibitor	**phase I/II** (NCT01647789)
ARN-509 [85]	SM	AR antagonist	**phase I/II** (NCT01822041, NCT01171898, NCT01792687[AP], NCT01790126/[G])
ODM-201 [86]	SM	AR antagonist	**phase I/II** (NCT01429064, NCT01784757, NCT01317641)
AZD-3514	SM	AR mRNA antagonist	**Phase I** (NCT01337518)
EZN-4176 [87]	AO	down-regulation of AR mRNA	**Phase I** (NCT01337518)
OGX-427 [88]	AO	heat shock protein 27 inhibitor	**Phase I/II** (NCT00487786/[D], NCT01120470[P], NCT01681433[AP])
Cabozantinib (XL184) [89]	SM	dual inhibitor of VEGFR/MET Angiogenesis inhibition	**Phase I/II** (NCT01428219, NCT01834651, NCT01599793, NCT01703065, NCT01630590[G], NCT01812668, NCT01683994[DP], NCT01347788, NCT01574937[A], NCT00940225) **Phase III** (NCT01605227, NCT01522443)
Dasatinib [90]	SM	Src tyrosine kinase inhibitor	**Phase I/II** (NCT00570700, NCT00918385, NCT01260688[C], NCT01685125[AP], NCT00385580, NCT00385580#, NCT00936975, NCT01254864[AP], NCT00439270[D]#, NCT01826838[R])
Cediranib [91] (AZD2171, Recentin)	SM	VEGFR tyrosine kinase inhibitor	**Phase I/II** (NCT01260688/[Da], NCT00527124 [DP], NCT00502164#, NCT00436956)
Sorafenib [92] (Nexavar, BAY43-9006)	SM	inhibitor of VEGFR, PDGFR and Raf kinase	**Phase I/II** (NCT00466752#, NCT00090545#, NCT00405210[D]#, NCT00619996[D]#, NCT00424385[I]#, NCT00093457#, NCT00430235[B]#, NCT00414388#, NCT00703638[PeCp]#)
Imatinib [93] (Gleevec, Glivec, STI571)	SM	BCR-Abl inhibitor	**Phase I/II** (NCT00080678[D]#, NCT00251225[D]#, NCT00038194[D]#, NCT00084825[D]#, NCT00427999#, NCT01316458#, NCT00171912#)
Sunitinib [94] (SU11248)	SM	inhibitor of VEGFR / PDGFR	**Phase I/II** (NCT00879619[DP], NCT00299741#, NCT00137436[DP]#, NCT00790595#, NCT00672594, NCT00631527[GR]#, NCT00879619[DP], NCT00748358, NCT00734851[DR], NCT00795171[D], NCT01254864[AP], NCT00550810, NCT00599313)
Aflibercept (AVE0005)	P	VEGF trap, anti-angiogensis	**Phase III** (NCT00519285[D]#)
TH-302 [79]	SM	hypoxia activated cytotoxicity	**Phase I/II** (NCT00743379)
Carboplatin [95]	PtC	cytotoxicity; inhibiting DNA synthesis	**Phase I/II** (NCT01051570[EP], NCT00973882[Ep]#, NCT00049257[Pt]#, NCT01505868[Ct], NCT00017576[Ca]#, NCT00514540[D]#, NCT00134706[D]#, CT00005627 [DEs]#, NCT00183924[DEs]#, NCT00016913, NCT00193193[EsPt]#, NCT01558492[Pt], NCT00003690[Fp]#, NCT00675545[D]#, NCT00005810[DEsFg]#)
Oxaliplatin [96]	PtC	cytotoxicity; inhibiting DNA synthesis	**Phase I/II** (NCT01338792[Pe]#, NCT00260611[D]#, NCT01487720[Gm])
Satraplatin [97]	PtC	cytotoxicity; inhibiting DNA synthesis	**Phase II** (NCT00499694[Bv], NCT00634647[P]) **Phase III** (NCT00069745[P]#)
Bevacizumab [98]	AB	angiogenesis inhibitor	**Phase II** (NCT00349557[GR]#, NCT00478413, NCT00776594[BG], NCT00658697[BDG], NCT00027599[S]#, NCT00942578[DLP]#, NCT00499694[Sp], NCT00089609[DPT], NCT00574769[DEl], NCT00321646[D]#, NCT00348998[BGR]) **Phase III** (NCT00110214[DP]#, NCT00942331[CpGm])
PROSTVAC-VF [80] (Prostvac)	V	immunotherapy recombinant vaccinia virus expresses PSA	**Phase I/II** (NCT00450463[F], NCT00078585, NCT00108732, NCT00113984, NCT00096551#, NCT00001382#, NCT00045227#, NCT00020254#, NCT00003871#, NCT00004029#) **Phase III** (NCT01322490)
Ipilimumab [82] (MDX010, Yervoy, BMS-734016, anti-CTLA4)	AB	immunotherapy anti-cytotoxic T lymphocyte-associated receptor 4 antibody	**Phase I/II** (NCT01530984, NCT01377389[G], NCT01194271[G], NCT01688492, NCT01498978, NCT00323882, NCT01832870[S], NCT00064129, NCT01804465, NCT00170157#, NCT00050596/[D]#, NCT00113984#) **Phase III** (NCT00861614, NCT01057810)
Nivolumab [83] (MDX1106, BMS-936558)	AB	immunotherapy anti programmed cell death protein 1 antibody	**Phase I/II** (NCT00441337#)
Custirsen [99] (OGX-011)	AO	clusterin inhibitor	**Phase I/II** (NCT00327340[D]#, NCT00054106[GF]#, NCT00258388[DP]#, NCT00471432[D]#) **Phase III** (NCT01578655[CtP], NCT01083615[Ct], NCT01188187[DP]#)
Zibotentan [100] (ZD4054)	SM	endothelin-A receptor antagonist	**Phase I/II** (NCT00090363#, NCT00055471#, NCT00314782[D]#, NCT01168141) **Phase III** (NCT00554229#, NCT00626548, NCT00617669[D]#)
Atrasentan [101]	SM	endothelin-A receptor antagonist	**Phase I/II** (NCT00181558[Z]#, NCT00038662#) **Phase III** (NCT00134056[DP], NCT00046943#, NCT00036556#, NCT00127478#)
Tasquinimod (ABR-215050) [102]	SM	anti-angiogenesis	**Phase I/II** (NCT01513733[CtP], NCT01732549, NCT00560482) **Phase III** (NCT01234311)

a For the structures of small molecules, see Scheme 2. For more information, see references noted;

b SM: small molecule; AO: antisense oligonucleotide; P: protein; AB: antibody; V: vaccine; PtC: platinum complex; PA: peptide analogue;

c VEGFR: vascular endothelial growth factor receptor; PDGFR: platelet-derived growth factor receptor;

d in combination with [A]: Abiraterone; [B]: Bicalutamide; [Bv]: Bevacizumab; [C]: Cediranib; [Ca]: Calcitriol; [Cp]: Cisplatin; [Ct]: Cabazitaxel; [D]: Docetaxel; [Da]: Dasatinib; [E]: Everolimus; [El]: Everolimus; [Ep]: Etoposide; [Es]: Estramustine; [F]: Flutamide; [Fg]: Filgrastim; [Fp]: Flavopiridol; [G]: GnRH analogue; [Gm]: Gemcitabine; [I]: Imatinib; [L]: Lenalidomide; [Op]: Oxaliplatin; [P]: Prednisone; [Pe]: Pemetrexed; [Pt]: Paclitaxel; [R]: radiation; [S]: Sipuleucel-T; [So]: Sorafenib; [Sp]: Satraplatin; [T]: Thalidomide; [Z]: Zoledronic acid;/: monotherapy or in combination; #: accomplished;

e registered in the EudraCT database (www.clinicaltrialsregister.eu).

## References

[1] Siegel R., Naishadham D., Jemal A. (2012). Cancer statistics, 2012. CA Cancer J. Clin.

[2] Siegel R., DeSantis C., Virgo K., Stein K., Mariotto A., Smith T., Cooper D., Gansler T., Lerro C., Fedewa S. (2012). Cancer treatment and survivorship statistics. CA Cancer J. Clin.

[3] Attard G., Reid A.H.M., Yap T.A., Raynaud F., Dowsett M., Settatree S., Barrett M., Parker C., Martins V., Folkerd E. (2008). Phase I clinical trial of a selective inhibitor of CYP17, abiraterone acetate, confirms that castration-resistant prostate cancer commonly remains hormone driven. J. Clin. Oncol.

[4] Weinzimer S.A., Gibson T.B., Collett-Solberg P.F., Khare A., Liu B., Cohen P. (2011). Transferrin is an insulin-like growth factor-binding protein-3 binding protein. J. Clin. Endocrinol. Metab.

[5] Geller J. (1993). Basis for hormonal management of advanced prostate cancer. Cancer.

[6] Titus M.A., Schell M.J., Lih F.B., Tomer K.B., Mohler J.L. (2005). Testosterone and dihydrotestosterone tissue levels in recurrent prostate cancer. Clin. Cancer Res.

[7] Hu Q., Negri M., Olgen S., Hartmann R.W. (2010). The role of fluorine substitution in biphenyl methylene imidazole type CYP17 inhibitors for the treatment of prostate carcinoma. ChemMedChem.

[8] Hu Q., Yin L., Jagusch C., Hille U.E., Hartmann R.W. (2010). Isopropylidene substitution increases activity and selectivity of biphenyl methylene 4-pyridine type CYP17 inhibitors. J. Med. Chem.

[9] Hu Q., Negri M., Jahn-Hoffmann K., Zhuang Y., Olgen S., Bartels M., Müller-Vieira U., Lauterbach T., Hartmann R.W. (2008). Synthesis, biological evaluation, and molecular modeling studies of methylene imidazole substituted biaryls as inhibitors of human 17α-hydroxylase-17,20-lyase (CYP17)—Part II: Core rigidification and influence of substituents at the methylene bridge. Bioorg. Med. Chem.

[10] Hille U.E., Hu Q., Vock C., Negri M., Bartels M., Mueller-Vieira U., Lauterbach T., Hartmann R.W. (2009). Novel CYP17 inhibitors: Synthesis, biological evaluation, structure-activity relationships and modeling of methoxy- and hydroxy-substituted methyleneimidazolyl biphenyls. Eur. J. Med. Chem.

[11] Pinto-Bazurco Mendieta M.A.E., Negri M., Hu Q., Hille U.E., Jagusch C., Jahn-Hoffmann K., Müller-Vieira U., Schmidt D., Lauterbach T., Hartmann R.W. (2008). CYP17 inhibitors—Annulations of additional rings in methylene imidazole substituted biphenyls: Synthesis, biological evaluation and molecular modeling. Arch. Pharm.

[12] Hille U.E., Hu Q., Pinto-Bazurco Mendieta M.A.E., Bartels M., Vock C.A., Lauterbach T., Hartmann R.W. (2009). Steroidogenic cytochrome P450 (CYP) enzymes as drug targets: Combining substructures of known CYP inhibitors leads to compounds with different inhibitory profile. C. R. Chim.

[13] Jagusch C., Negri M., Hille U.E., Hu Q., Bartels M., Jahn-Hoffmann K., Pinto-Bazurco Mendieta M.A.E., Rodenwaldt B., Müller-Vieira U., Schmidt D. (2008). Synthesis, biological evaluation and molecular modeling studies of methyleneimidazole substituted biaryls as inhibitors of human 17α-hydroxylase-17,20-lyase (CYP17)—Part I: Heterocyclic modifications of the core structure. Bioorg. Med. Chem.

[14] Krug S.J., Hu Q., Hartmann R.W. (2013). Hits identified in library screening demonstrate selective CYP17A1 lyase inhibition. J. Steroid Biochem. Mol. Biol.

[15] Abadi A.H., Abou-Seri S.M., Hu Q., Negri M., Hartmann R.W. (2012). Synthesis and biological evaluation of imidazolylmethylacridones as cytochrome P-450 enzymes inhibitors. Med. Chem. Commun.

[16] Gobbi S., Cavalli A., Rampa A., Belluti F., Piazzi L., Paluszcak A., Hartmann R.W., Recanatini M., Bisi A. (2006). Lead optimization providing a series of flavone derivatives as potent nonsteroidal inhibitors of the cytochrome P450 aromatase enzyme. J. Med. Chem.

[17] Leze M.P., Palusczak A., Hartmann R.W., le Borgne M. (2008). Synthesis of 6- or 4-functionalized indoles via a reductive cyclization approach and evaluation as aromatase inhibitors. Bioorg. Med. Chem. Lett.

[18] Al-Soud Y.A., Heydel M., Hartmann R.W. (2011). Design and synthesis of 1,3,5-trisubstituted 1,2,4-triazoles as CYP enzyme inhibitors. Tetrahedron Lett.

[19] Yin L., Hu Q. (2013). Drug discovery for breast cancer and coinstantaneous cardiovascular disease: What is the future?. Future Med. Chem.

[20] Yin L., Lucas S., Maurer F., Kazmaier U., Hu Q., Hartmann R.W. (2012). Novel imidazol-1-ylmethyl substituted 1,2,5,6-tetrahydropyrrolo[3,2,1-*ij*]quinolin-4-ones as potent and selective CYP11B1 inhibitors for the treatment of Cushing’s syndrome. J. Med. Chem.

[21] Emmerich J., Hu Q., Hanke N., Hartmann R.W. (2013). Cushing’s syndrome: Development of highly potent and selective CYP11B1 inhibitors of the (pyridylmethyl)pyridine type. J. Med. Chem.

[22] Gobbi S., Hu Q., Negri M., Zimmer C., Belluti F., Rampa A., Hartmann R.W., Bisi A. (2013). Modulation of cytochromes P450 with xanthone-based molecules: From aromatase to aldosterone synthase and steroid 11β-hydroxylase inhibition. J. Med. Chem.

[23] Yin L., Hu Q., Hartmann R.W. (2012). 3-Pyridinyl substituted aliphatic cycles as CYP11B2 inhibitors: Aromaticity abolishment of the core significantly increased selectivity over CYP1A2. PLoS One.

[24] Grombein C.M., Hu Q., Heim R., Hartmann R.W. (2013). Unexpected results of a SN,Ar-reaction—A novel synthetic approach to 1-arylthio- naphthalen-2-ols. Adv. Synth. Catal..

[25] Hu Q., Yin L., Hartmann R.W. (2013). Novel heterocycle substituted 4,5-dihydro-[1,2,4]triazolo[4,3-a]quinolines as potent and selective aldosterone synthase inhibitors for the treatment of aldosterone-related cardiovascular diseases. J. Med. Chem..

[26] Grombein C.M., Hu Q., Heim R., Rau S., Zimmer C., Hartmann R.W. (2013). 1-Phenylsulfinyl-3- (pyridin-3-yl)naphthalen-2-ols: A new class of potent and selective aldosterone synthase inhibitors. J. Med. Chem..

[27] Yin L., Hu Q., Hartmann R.W. (2013). Novel pyridyl or isoquinolinyl substituted indolines and indoles as potent and selective aldosterone synthase inhibitors. J. Med. Chem..

[28] Hu Q., Yin L., Hartmann R.W. (2012). Selective dual inhibitors of CYP19 and CYP11B2: Targeting cardiovascular diseases hiding in the shadow of breast cancer. J. Med. Chem.

[29] Yin L., Hu Q., Hartmann R.W. (2013). Tetrahydropyrroloquinolinone type dual inhibitors of aromatase/aldosterone synthase as a novel strategy for breast cancer patients with elevated cardiovascular risks. J. Med. Chem.

[30] Potter G.A., Banie S.E., Jarman M., Rowlands M.G. (1995). Novel steroidal inhibitors of human cytochrome P450_17α_ (l7α-hydroxylase-C_l7,20_-lyase): Potential agents for the treatment of prostatic cancer. J. Med. Chem.

[31] Yin L., Hu Q. (2013). CYP17 inhibitors: From promiscuous abiraterone to selective C17-20 lyase inhibitors and multi-targeting agents. Nat. Rev. Urol..

[32] Soifer H.S., Souleimanian N., Wu S., Voskresenskiy A.M., Collak F.K., Cinar B., Stein C.A. (2012). Direct regulation of androgen receptor activity by potent CYP17 inhibitors in prostate cancer cells. J. Bio. Chem.

[33] Zytiga prescribing information.

[34] Ryan C.J., Smith M.R., Fong L., Rosenberg J.E., Kantoff P., Raynaud F., Martins V., Lee G., Kheoh T., Kim J. (2010). Phase I clinical trial of the CYP17 inhibitor abiraterone acetate demonstrating clinical activity in patients with castration-resistant prostate cancer who received prior ketoconazole therapy. J. Clin. Oncol.

[35] Acharya M., Gonzalez M., Mannens G., de Vries R., Lopez C., Griffin T., Tran N. (2013). A phase I, open-label, single-dose, mass balance study of 14C-labeled abiraterone acetate in healthy male subjects. Xenobiotica.

[36] Efstathiou E., Titus M., Tsavachidou D., Tzelepi V., Wen S., Hoang A., Molina A., Chieffo N., Smith L.A., Karlou M. (2012). Effects of abiraterone acetate on androgen signaling in castrate-resistant prostate cancer in bone. J. Clin. Oncol.

[37] Attard G., Reid A.H.M., A’Hern R., Parker C., Oommen N.B., Folkerd E., Messiou C., Molife L.R., Maier G., Thompson E. (2009). Selective inhibition of CYP17 with abiraterone acetate is highly active in the treatment of castration-resistant prostate cancer. J. Clin. Oncol.

[38] Ryan C.J., Shah S., Efstathiou E., Smith M.R., Taplin M.E., Bubley G.J., Logothetis C.J., Kheoh T., Kilian C., Haqq C.M. (2011). Phase II study of abiraterone acetate in chemotherapy-naïve metastatic castration-resistant prostate cancer displaying bone flare discordant with serologic response. Clin. Cancer Res.

[39] Danila D.C., Morris M.J., de Bono J.S., Ryan C.S., Denmeade S.R., Smith M.R., Taplin M.E., Bubley G.J., Kheoh T., Haqq C. (2010). Phase II multicenter study of abiraterone acetate plus prednisone therapy in patients with docetaxel-treated castration-resistant prostate cancer. J. Clin. Oncol.

[40] Reid A.H.M., Attard G., Danila D.C., Oommen N.B., Olmos D., Fong P.C., Molife L.R., Hunt J., Messiou C., Parker C. (2010). Significant and sustained antitumor activity in postdocetaxel, castration-resistant prostate cancer with the CYP17 inhibitor abiraterone acetate. J. Clin. Oncol.

[41] Ryan C.J., Smith M.R., de Bono J.S., Molina A., Logothetis C.J., de Souza P., Fizazi K., Mainwaring P., Piulats J.M., Ng S. (2013). Abiraterone in metastatic prostate cancer without previous chemotherapy. N. Engl. J. Med.

[42] Logothetis C.J., Basch E., Molina A., Fizazi K., North S.A., Chi K.N., Jones R.J., Goodman O.B., Mainwaring P.N., Sternberg C.N. (2012). Effect of abiraterone acetate and prednisone compared with placebo and prednisone on pain control and skeletal-related events in patients with metastatic castration-resistant prostate cancer: Exploratory analysis of data from the COU-AA-301 randomised trial. Lancet Oncol.

[43] Sternberg C.N., Molina A., North S., Mainwaring P., Fizazi K., Hao Y., Rothman M., Gagnon D.D., Kheoh T., Haqq C.M. (2012). Effect of abiraterone acetate on fatigue in patients with metastatic castration-resistant prostate cancer after docetaxel chemotherapy. Ann. Oncol.

[44] De Bono J.S., Logothetis C.J., Molina A., Fizazi K., North S., Chu L., Chi K.N., Jones R.J., Goodman O.B., Saad F. (2011). Abiraterone and increased survival in metastatic prostate cancer. N. Engl. J. Med..

[45] Fizazi K., Scher H.I., Molina A., Logothetis C.J., Chi K.N., Jones R.J., Staffurth J.N., North S., Vogelzang N.J., Saad F. (2012). Abiraterone acetate for treatment of metastatic castration-resistant prostate cancer: Final overall survival analysis of the COU-AA-301 randomised, double-blind, placebo-controlled phase 3 study. Lancet Oncol.

[46] Zagar Y., Chaumaz G., Lieberherr M. (2004). Signaling cross-talk from Gbeta4 subunit to Elk-1 in the rapid action of androgens. J. Bio. Chem.

[47] Kampa M., Papakonstanti E.A., Hatzoglou A., Stathopoulos E.N., Stournaras C., Castanas E. (2002). The human prostate cancer cell line LNCaP bears functional membrane testosterone receptors, which increase PSA secretion and modify actin cytoskeleton. FASEB J.

[48] Chen C.D., Welsbie D.S., Tran C., Baek S.H., Chen R., Vessella R., Rosenfeld M.G., Sawyers C.L. (2004). Molecular determinants of resistance to antiandrogen therapy. Nat. Med.

[49] Small E.J., Srinivas S. (1995). The antiandrogen withdrawal syndrome—Experience in a large cohort of unselected patients with advanced prostate cancer. Cancer.

[50] Taplin M.E., Bubley G.J., Shuster T.D., Frantz M.E., Spooner A.E., Ogata G.K., Keer H.N., Balk S.P. (1995). Mutation of the androgen-receptor gene in metastatic androgen-independent prostate cancer. N. Engl. J. Med.

[51] Hara T., Miyazaki J., Araki H., Yamaoka M., Kanzaki N., Kusaka M., Miyamoto M. (2003). Novel mutations of androgen receptor: A possible mechanism of bicalutamide withdrawal syndrome. Cancer Res.

[52] Tran C., Ouk S., Clegg N.J., Chen Y., Watson P.A., Arora V., Wongvipat J., Smith-Jones P.M., Yoo D., Kwon A. (2009). Development of a second-generation antiandrogen for treatment of advanced prostate cancer. Science.

[53] Belikov S., Öberg C., Jääskeläinen T., Rahkama V., Palvimo J.J., Wrange Ö. (2013). FoxA1 corrupts the antiandrogenic effect of bicalutamide but only weakly attenuates the effect of MDV3100 (Enzalutamide™). Mol. Cell. Endocrinol..

[54] Scher H.I., Beer T.M., Higano C.S., Anand A., Taplin M.E., Efstathiou E., Rathkopf D., Shelkey J., Yu E.Y., Alumkal J. (2010). Antitumour activity of MDV3100 in castration- resistant prostate cancer: A phase 1–2 study. Lancet.

[55] Scher H.I., Fizazi K., Saad F., Taplin M.E., Sternberg C.N., Miller K., de Wit R., Mulders P., Chi K.N., Shore N.D. (2012). Increased survival with enzalutamide in prostate cancer after chemotherapy. N. Engl. J. Med.

[56] Tannock I.F., de Wit R., Berry W.R., Horti J., Pluzanska A., Chi K.N., Oudard S., Théodore C., James N.D., Turesson I. (2004). Docetaxel plus prednisone or mitoxantrone plus prednisone for advanced prostate cancer. N. Engl. J. Med.

[57] De Bono J.S., Oudard S., Ozguroglu M., Hansen S., Machiels J.P., Kocak I., Gravis G., Bodrogi I., Mackenzie M.J., Shen L. (2010). Prednisone plus cabazitaxel or mitoxantrone for metastatic castration-resistant prostate cancer progressing after docetaxel treatment: A randomised open-label trial. Lancet.

[58] Mita A.C., Denis L.J., Rowinsky E.K., de Bono J.S., Goetz A.D., Ochoa L., Forouzesh B., Beeram M., Patnaik A., Molpus K. (2009). Phase I and pharmacokinetic study of XRP6258 (RPR 116258A), a novel taxane, administered as a 1-hour infusion every 3 weeks in patients with advanced solid tumors. Clin. Cancer Res.

[59] Kearns B., Lloyd J.M., Stevenson M., Littlewood C. (2013). Cabazitaxel for the second-line treatment of metastatic hormone-refractory prostate cancer: A NICE single technology appraisal. Pharmacoeconomics.

[60] Provenge—FDA full prescribing information.

[61] Kantoff P.W., Higano C.S., Shore N.D., Berger E.R., Small E.J., Penson D.F., Redfern C.H., Ferrari A.C., Dreicer R., Sims R.B. (2010). Sipuleucel-T immunotherapy for castration-resistant prostate cancer. N. Engl. J. Med.

[62] Higano C.S., Schellhammer P.F., Small E.J., Burch P.A., Nemunaitis J., Yuh L., Provost N., Frohlich M.W. (2009). Integrated data from 2 randomized, double-blind, placebo-controlled, phase 3 trials of active cellular immunotherapy with sipuleucel-T in advanced prostate cancer. Cancer.

[63] Small E.J., Schellhammer P.F., Higano C.S., Redfern C.H., Nemunaitis J.J., Valone F.H., Verjee S.S., Jones L.A., Hershberg R.M. (2006). Placebo-controlled phase III trial of immunologic therapy with sipuleucel-T (APC8015) in patients with metastatic, asymptomatic hormone refractory prostate cancer. J. Clin. Oncol.

[64] Saylor P.J., Lee R.J., Smith M.R. (2011). Emerging therapies to prevent skeletal morbidity in men with prostate cancer. J. Clin. Oncol.

[65] Pfeilschifter J., Mundy G.R. (1987). Modulation of type beta transforming growth factor activity in bone cultures by osteotropic hormones. Proc. Natl. Acad. Sci. USA.

[66] Brown J.M., Corey E., Lee Z.D., True L.D., Yun T.J., Tondravi M., Vessella R.L. (2001). Osteoprotegerin and rank ligand expression in prostate cancer. Urology.

[67] Smith M.R., Egerdie B., Hernández T.N., Feldman R., Tammela T.L., Saad F., Heracek J., Szwedowski M., Ke C., Kupic A. (2009). Denosumab in men receiving androgen-deprivation therapy for prostate cancer. N. Engl. J. Med..

[68] Fizazi K., Carducci M., Smith M., Damião R., Brown J., Karsh L., Milecki P., Shore N., Rader M., Wang H. (2011). Denosumab *versus* zoledronic acid for treatment of bone metastases in men with castration-resistant prostate cancer: A randomised, double-blind study. Lancet.

[69] Fizazi K., Bosserman L., Gao G., Skacel T., Markus R. (2009). Denosumab treatment of prostate cancer with bone metastases and increased urine N-telopeptide levels after therapy with intravenous bisphosphonates: Results of a randomized phase II trial. J. Urol.

[70] Smith M.R., Saad F., Coleman R., Shore N., Fizazi K., Tombal B., Miller K., Sieber P., Karsh L., Damião R. (2012). Denosumab and bone-metastasis-free survival in men with castration-resistant prostate cancer: Results of a phase 3, randomised, placebo-controlled trial. Lancet.

[71] Nilsson S., Franzén L., Parker C., Tyrrell C., Blom R., Tennvall J., Lennernäs B., Petersson U., Johannessen D.C., Sokal M. (2007). Bone-targeted radium-223 in symptomatic, hormonerefractory prostate cancer: A randomised, multicentre, placebo-controlled phase II study. Lancet Oncol.

[72] Xofigo full prescribing information.

[73] Lassmann M., Nosske D. (2013). Dosimetry of ^223^Ra-chloride: Dose to normal organs and tissues. Eur. J. Nucl. Med. Mol. Imaging.

[74] Vogelzang N.J., Helle S.I., Johannessen D.C., O’Sullivan J.M., Garcia-Vargas J.E., O’Bryan-Tear C.G., Shan M., Parker C. (2013). Efficacy and safety of radium-233 dichloride (Ra-233) in castration-resistant prostate cancer (CRPC) patients with bone metastases who did or did not receive prior docetaxel (D) in the phase III ALSYMPCA trial. J. Clin. Oncol..

[75] Vasaitis T., Belosay A., Schayowitz A., Khandelwal A., Chopra P., Gediya K.Z., Guo L., Fang H.B., Njar V.C.O., Brodie A.M.H. (2008). Androgen receptor inactivation contributes to antitumor efficacy of 17α-hydroxylase/17,20-lyase inhibitor 3β-hydroxy-17-(1H-benzimidazole-1-yl)-androsta- 5,16-diene in prostate cancer. Mol. Cancer Ther.

[76] American Association for Cancer Research Web Page Early Clinical Data Show Galeterone Safe, Effective against Prostate Cancer.

[77] Eisner J.R., Abbott D.H., Bird I.M., Rafferty S.W., Moore W.R., Schotzinger R.J. (2012). Assessment of steroid hormones upstream of P450c17 (CYP17) in chemically castrate male rhesus monkeys following treatment with the CYP17 inhibitors VT-464 and abiraterone acetate (AA). Endocr. Rev..

[78] Abbott D.H., Eisner J.R., Bird I.M., Rafferty S.W., Moore W.R., Schotzinger R.J. (2012). Plasma steroid concentrations in male rhesus monkeys following treatment with the P450c17 (CYP17) inhibitors VT-464 and abiraterone acetate: A comparison to human 17,20-Lyase (lyase) and combined lyase/17α-hydroxylase (hydroxylase) deficiencies. Endocr. Rev..

[79] Liu Q., Sun J.D., Wang J., Ahluwalia D., Baker A.F., Cranmer L.D., Ferraro D., Wang Y., Duan J.X., Ammons W.S. (2012). TH-302, a hypoxia-activated prodrug with broad *in vivo* preclinical combination therapy efficacy: Optimization of dosing regimens and schedules. Cancer Chemother. Pharmacol.

[80] Gulley J.L., Madan R.A., Stein W.D., Wilkerson J., Dahut W.L., Heery C.R., Schlom J., Wilding G., DiPaola R.S. (2013). Effect of PSA-tricom, a pox-viral vaccine in prostate cancer (PCa), on tumor growth rates within 80 days after initiation in nonmetastatic PCa. J. Clin. Oncol..

[81] Drake C.G., Jaffee E., Pardoll D.M. (2006). Mechanisms of immune evasion by tumors. Adv. Immunol.

[82] Madan R.A., Mohebtash M., Arlen P.M., Vergati M., Rauckhorst M., Steinberg S.M., Tsang K.Y., Poole D.J., Parnes H.L., Wright J.J. (2012). Ipilimumab and a poxviral vaccine targeting prostate-specific antigen in metastatic castration-resistant prostate cancer: A phase 1 dose-escalation trial. Lancet Oncol.

[83] Topalian S.L., Hodi F.S., Brahmer J.R., Gettinger S.N., Smith D.C., McDermott D.F., Powderly J.D., Carvajal R.D., Sosman J.A., Atkins M.B. (2012). Safety, activity, and immune correlates of anti-PD-1 antibody in cancer. N. Engl. J. Med.

[84] Agus D.B., Stadler W.M., Shevrin D.H., Hart L., MacVicar G.R., Hamid O., Hainsworth J.D., Gross M.E., Wang J., Webb I.J. (2012). Safety, efficacy, and pharmacodynamics of the investigational agent orteronel (TAK-700) in metastatic castration-resistant prostate cancer (mCRPC): Updated data from a phase I/II study. J. Clin. Oncol..

[85] Clegg N.J., Wongvipat J., Joseph J.D., Tran C., Ouk S., Dilhas A., Chen Y., Grillot K., Bischoff E.D., Cai L. (2012). ARN-509: A novel antiandrogen for prostate cancer treatment. Cancer Res.

[86] Massard C., James N., Culine S., Jones R., Vuorela A., Mustonen M., Fizazi K. ARADES Trial: A First-in-Man, Open-Label, Phase I/II Safety, Pharmacokinetic, and Proof-of-Concept Study of ODM-201 in Patients (pts) with Progressive Metastatic Castration-Resistant Prostate Cancer (mCRPC).

[87] Zhang Y., Castaneda S., Dumble M., Wang M., Mileski M., Qu Z., Kim S., Shi V., Kraft P., Gao Y. (2011). Reduced expression of the androgen receptor by third generation of antisense shows antitumor activity in models of prostate cancer. Mol. Cancer Ther..

[88] Chi K., Yu E.Y., Ellard S., Hotte S.J., Gingerich J.R., Joshua A.M., Gleave M.E. A Randomized Phase II Study of OGX-427 plus Prednisone (P) *vs.* P alone in Patients (pts) with Metastatic Castration Resistance Prostate Cancer (CRPC).

[89] Smith D.C., Smith M.R., Sweeney C., Elfiky A.A., Logothetis C., Corn P.G., Vogelzang N.J., Small E.J., Harzstark A.L., Gordon M.S. (2013). Cabozantinib in patients with advanced prostate cancer: Results of a phase II randomized discontinuation trial. J. Clin. Oncol.

[90] Araujo J.C., Mathew P., Armstrong A.J., Braud E.L., Posadas E., Lonberg M., Gallick G.E., Trudel G.C., Paliwal P., Agrawal S. (2012). Dasatinib combined with docetaxel for castration-resistant prostate cancer: Results from a phase 1–2 study. Cancer.

[91] Dahut W.L., Madan R.A., Karakunnel J.J., Adelberg D., Gulley J.L., Turkbey I.B., Chau C.H., Spencer S.D., Mulquin M., Wright J. (2013). Phase II clinical trial of cediranib in patients with metastatic castration-resistant prostate cancer. BJU Int..

[92] Mardjuadi F., Medioni J., Kerger J., D’Hondt L., Canon J.L., Duck L., Musuamba F., Oudard S., Clausse M., Moxhon A. (2012). Phase I study of sorafenib in combination with docetaxel and prednisone in chemo-naïve patients with metastatic castration-resistant prostate cancer. Cancer Chemother. Pharmacol.

[93] Nabhan C., Villines D., Valdez T.V., Tolzien K., Lestingi T.M., Bitran J.D., Christner S.M., Egorin M.J., Beumer J.H. (2012). Phase I study investigating the safety and feasibility of combining imatinib mesylate (Gleevec) with sorafenib in patients with refractory castration-resistant prostate cancer. Br. J. Cancer.

[94] Saylor P.J., Mahmood U., Kunawudhi A., Smith M.R., Palmer E.L., Michaelson M.D. (2012). Multitargeted tyrosine kinase inhibition produces discordant changes between ^99m^Tc-MDP bone scans and other disease biomarkers: analysis of a phase II study of sunitinib for metastatic castration-resistant prostate cancer. J. Nucl. Med.

[95] Kentepozidis N., Soultati A., Giassas S., Vardakis N., Kalykaki A., Kotsakis A., Papadimitraki E., Pantazopoulos N., Bozionellou V., Georgoulias V. (2012). Paclitaxel in combination with carboplatin as salvage treatment in patients with castration-resistant prostate cancer: A Hellenic oncology research group multicenter phase II study. Cancer Chemother. Pharmacol.

[96] Gasent B.J.M., Giner M.V., Giner-Bosch V., Cerezuela F.P., Alberola C.V. (2011). Phase II trial of oxaliplatin and capecitabine after progression to first-line chemotherapy in androgen-independent prostate cancer patients. Am. J. Clin. Oncol.

[97] Cetnar J., Wilding G., McNeel D., Loconte N.K., McFarland T.A., Eickhoff J., Liu G. (2013). A phase 1/1b study of satraplatin (JM-216) in combination with docetaxel in patients with advanced solid tumors and metastatic castrate-resistant prostate cancer. Urol. Oncol.

[98] Kelly W.K., Halabi S., Carducci M., George D., Mahoney J.F., Stadler W.M., Morris M., Kantoff P., Monk J.P., Kaplan E. (2012). Randomized, double-blind, placebo-controlled phase III trial comparing docetaxel and prednisone with or without bevacizumab in men with metastatic castration-resistant prostate cancer: CALGB 90401. J. Clin. Oncol.

[99] Saad F., Hotte S., North S., Eigl B., Chi K., Czaykowski P., Wood L., Pollak M., Berry S., Lattouf J.B. (2011). Randomized phase II trial of Custirsen (OGX-011) in combination with docetaxel or mitoxantrone as second-line therapy in patients with metastatic castrate-resistant prostate cancer progressing after first-line docetaxel: CUOG trial P-06c. Clin. Cancer Res.

[100] Fizazi K.S., Higano C.S., Nelson J.B., Gleave M., Miller K., Morris T., Nathan F.E., McIntosh S., Pemberton K., Moul J.W. (2013). Phase III, randomized, placebo-controlled study of docetaxel in combination with zibotentan in patients with metastatic castration-resistant prostate cancer. J. Clin. Oncol.

[101] Armstrong A.J., Creel P., Turnbull J., Moore C., Jaffe T.A., Haley S., Petros W., Yenser S., Gockerman J.P., Sleep D. (2008). A phase I–II study of docetaxel and atrasentan in men with castration-resistant metastatic prostate cancer. Clin. Cancer Res.

[102] Pili R., Häggman M., Stadler W.M., Gingrich J.R., Assikis V.J., Björk A., Nordle O., Forsberg G., Carducci M.A., Armstrong A.J. (2011). Phase II randomized, double-blind, placebo-controlled study of tasquinimod in men with minimally symptomatic metastatic castrate-resistant prostate cancer. J. Clin. Oncol.

[103] Zhao X.Y., Malloy P.J., Krishnan A.V., Swami S., Navone N.M., Peehl D.M., Feldman D. (2000). Glucocorticoids can promote androgen-independent growth of prostate cancer cells through a mutated androgen receptor. Nat. Med.

[104] Hu Q., Jagusch C., Hille U.E., Haupenthal J., Hartmann R.W. (2010). Replacement of imidazolyl by pyridyl in biphenyl methylenes results in selective CYP17 and dual CYP17/CYP11B1 inhibitors for the treatment of prostate cancer. J. Med. Chem.

[105] Efstathiou J.A., Bae K., Shipley W.U., Hanks G.E., Pilepich M.V., Sandler H.M., Smith M.R. (2008). Cardiovascular mortality after androgen deprivation therapy for locally advanced prostate cancer: RTOG 85-31. J. Clin. Oncol.

[106] Hu Q., Pinto-Bazurco Mendieta M.A.E., Hartmann R.W. (2013). Highly potent and selective non-steroidal dual Inhibitors of CYP17/CYP11B2 for the treatment of prostate cancer in reducing cardiovascular complications. J. Med. Chem..

[107] Mostaghel E.A., Marck B.T., Plymate S.R., Vessella R.L., Balk S., Matsumoto A.M., Nelson P.S., Montgomery R.B. (2011). Resistance to CYP17A1 inhibition with abiraterone in castration-resistant prostate cancer: induction of steroidogenesis and androgen receptor splice variants. Clin. Cancer Res.

[108] Li Y., Chan S.C., Brand L.J., Hwang T.H., Silverstein K.A., Dehm S.M. (2013). Androgen receptor splice variants mediate enzalutamide resistance in castration-resistant prostate cancer cell lines. Cancer Res.

